# The Exponential Diophantine Equation 2^*x*^ + *b* ^*y*^ = *c* ^*z*^

**DOI:** 10.1155/2014/401816

**Published:** 2014-05-18

**Authors:** Yahui Yu, Xiaoxue Li

**Affiliations:** ^1^Luoyang Institute of Science and Technology, Luoyang, Henan 471023, China; ^2^School of Mathematics, Northwest University, Xi'an, Shaanxi 710127, China

## Abstract

Let *b* and *c* be fixed coprime odd positive integers with min{*b*, *c*} > 1. In this paper, a classification of all positive integer solutions (*x*, *y*, *z*) of the equation 2^*x*^ + *b* ^*y*^ = *c* ^*z*^ is given. Further, by an elementary approach, we prove that if *c* = *b* + 2, then the equation has only the positive integer solution (*x*, *y*, *z*) = (1,1, 1), except for (*b*, *x*, *y*, *z*) = (89,13,1, 2) and (2^*r*^ − 1, *r* + 2,2, 2), where *r* is a positive integer with *r* ≥ 2.

## 1. Introduction

Let *N* be the set of all positive integers. Let *a*, *b*, *c* be fixed coprime positive integers with min⁡{*x*, *y*, *z*} > 1. In recent years, the solutions (*x*, *y*, *z*) of the equation

(1) $$\begin{array}{c} a^{x}+b^{y}=c^{z},\;x,y,z\in N \end{array}$$

have been investigated in many papers (see [1–3] and its references). In this paper we deal with (1) for the case that *a* = 2. Then (1) can be rewritten as

(2) $$\begin{array}{c} 2^{x}+b^{y}=c^{z},\;x,y,z\in N, \end{array}$$

where *b* and *c* are fixed coprime odd positive integers with min⁡{*b*, *c*} > 1. We will give a classification of all solutions (*x*, *y*, *z*) of (2) as follows.

**Theorem 1**

Every solution (*x*, *y*, *z*) of (2) satisfies one of the following types:

- (*b*, *c*, *x*, *y*, *z*) = (7,3, 5,2, 4);
- (*b*, *c*, *x*, *y*, *z*) = (2^*r*^ − 1, 2^*r*^ + 1, *r* + 2,2, 2), where *r* is a positive integer with *r* ≥ 2;
- (*b*, *c*, *x*, *y*, *z*) = (5,3, 1,2, 3);
- (*b*, *c*, *x*, *y*, *z*) = (11,5, 2,2, 3);
- 2 | *y* and *z* = 1;
- (*b*, *c*, *x*, *y*, *z*) = (17,71,7, 3,2);
- *x* = 1, *y* > 1, 2∤*y* and 2 | *z*;
- *x* > 1, *y* = 1, 2 | *z* and 2^*x*^ < *b* ^50/13^;
- 2∤*yz*.

Recently, Miyazaki and Togbé [4] showed that if  *b* ≥ 5 and *c* = *b* + 2, then (2) has only the solution (*x*, *y*, *z*) = (1,1, 1), except for (*b*, *x*, *y*, *z*) = (89,13,1, 2). However, there are some exceptional cases missing from the result of [4]. In this paper, by an elementary approach, we prove the following result.

**Corollary 2**

If *c* = *b* + 2, then (2) has only the solution (*x*, *y*, *z*) = (1,1, 1), except for (*b*, *x*, *y*, *z*) = (89,13,1, 2) and (2^*r*^ − 1, *r* + 2,2, 2), where *r* is a positive integer with *r* ≥ 2.

## 2. Preliminaries

**Lemma 3 (see [5, Formula 1.76])**

For any positive integer *n* and any complex numbers *α* and *β*, one has

(3) $$\begin{array}{c} \alpha^{n}+\beta^{n}=\overset{\left[n/2\right]}{\underset{i=0}{\sum }}\left[\begin{array}{c} n\\ i \end{array}\right]{\left(\alpha +\beta \right)}^{n-2i}{\left(-\alpha \beta \right)}^{i}, \end{array}$$

where [*n*/2] is the integer part of *n*/2;

(4) $$\begin{array}{c} \left[\begin{array}{c} n\\ i \end{array}\right]=\frac{\left(n-i-1\right)!n}{\left(n-2i\right)!i!},\;i=0,\ldots ,\left[\frac{n}{2}\right] \end{array}$$

are positive integers.

**Lemma 4 (see [6])**

Let *A* and *B* be coprime odd positive integers with min⁡{*A*, *B*} > 1. If the equation

(5) $$\begin{array}{c} Au^{2}-Bv^{2}=2,\;u,v\in N \end{array}$$

has solutions (*u*, *v*), then it has a unique solution (*u*_1_, *v*_1_) such that $u_{1}\sqrt{A}+v_{1}\sqrt{B}\leq u\sqrt{A}+v\sqrt{B}$, where (*u*, *v*) through all solutions of (5). The solution (*u*_1_, *v*_1_) is called the least solution of (5). Every solution (*u*, *v*) of (5) can be expressed as

(6) $$\begin{array}{c} \frac{u\sqrt{A}+v\sqrt{B}}{\sqrt{2}}={\left(\frac{u_{1}\sqrt{A}+v_{1}\sqrt{B}}{\sqrt{2}}\right)}^{n},\;n\in N,\;\;2∤n. \end{array}$$

Further, by (6), we have *u*_1_ | *u* and *v*_1_ | *v*.

**Lemma 5**

Equation (5) has no solutions (*u*, *v*) such that *u* > *u*_1_, *v* > *v*_1_, and every prime divisor of *u*/*u*_1_ and *v*/*v*_1_ divides *A* and *B*, respectively.

**Proof**

We now assume that (*u*, *v*) is a solution of (5) satisfying the hypothesis. Since *u* > *u*_1_, by Lemma 4, the (*u*_*n*_, *v*_*n*_) is all solutions of (5). Let

(7) $$\begin{array}{c} \alpha =\frac{u_{1}\sqrt{A}+v_{1}\sqrt{B}}{\sqrt{2}},\;\;\beta =\frac{u_{1}\sqrt{A}-v_{1}\sqrt{B}}{\sqrt{2}}. \end{array}$$

We get

(8) $$\begin{array}{c} \frac{u_{n}}{u_{1}}=\frac{\alpha^{n}-{\left(-\beta \right)}^{n}}{\alpha -\left(-\beta \right)},\;\;\frac{v_{n}}{v_{1}}=\frac{\alpha^{n}-{\left(\beta \right)}^{n}}{\alpha -\left(\beta \right)}, \end{array}$$

where *n* is odd. Numbers *α* and *β* are such that (*α*, −*β*) satisfy $x^{2}-\sqrt{2Bv_{1}^{2}}x+1=0$ and (*α*, *β*) satisfy $x^{2}-\sqrt{2Au_{1}^{2}}x+1=0$. Thus, {*u*_*n*_/*u*_1_}_*n*≥1_ and {*v*_*n*_/*v*_1_}_*n*≥1_ are the odd indexed subsequences of the two Lehmer sequences of roots (*α*, −*β*) and (*α*, *β*). Their discriminants are (*α* + *β*)^2^ = 2*Au*_1_^2^ and (*α* − *β*)^2^ = 2*Bv*_1_^2^, respectively. Saying that all prime factors of *u*_*n*_/*u*_1_ divide *A* implies that all primes of the *n*th term of a Lehmer sequence divide its discriminant. The same is true for *v*_*n*_/*v*_1_. Hence, *u*_*n*_/*u*_1_ and *v*_*n*_/*v*_1_ are terms of a Lehmer sequence of real roots lacking primitive divisors. By Table 2 in [7], this is possible only for *n* = 3,5. Even more, in the present case,

(9) $$\begin{array}{c} \frac{{\left(\alpha^{2}\right)}^{n}-{\left(\beta^{2}\right)}^{n}}{\alpha^{2}-\beta^{2}}=\frac{u_{n}v_{n}}{u_{1}v_{1}} \end{array}$$

is the *n*th term of the Lucas sequence of positive real roots (*α* ^2^, *β* ^2^) whose all prime factors divide its discriminant (*α* ^2^ − *β* ^2^)^2^ = 4*ABu*_1_^2^ *v*_2_^2^, and by Table 1 in [7] this is possible for *n* odd only if *n* = 3 or *n* = 5. Furthermore, when *n* = 5, we must have $\alpha^{2}=(1+\sqrt{5})/2$, but this is not possible since $\alpha =\sqrt{(1+\sqrt{5})/2}$ is not of the form $(u_{1}\sqrt{A}+v_{1}\sqrt{B})/\sqrt{2}$ for some positive integers *A* > 1, *B* > 1, *u*_1_ and *v*_1_. So, only *n* = 3 is possible. Now by some simple numerical computation for *u*_3_ and *v*_3_, we see that it is not possible that all prime factors of *u*_3_ and all prime factors of *v*_3_ divide* B*. Thus, Lemma 5 is proved.

**Lemma 6 (see [8])**

The equation

(10) $$\begin{array}{c} X^{2}+7=2^{n+2},\;X,n\in N \end{array}$$

has only the solutions (*X*, *n*) = (1,1), (3,2), (5,3), (11,5), and (181,13).

**Lemma 7 (see [9])**

Let *D* be an odd positive integer with *D* > 1. If (*X*, *n*) is a solution of the equation

(11) $$\begin{array}{c} X^{2}-D=2^{n},\;X,n\in N, \end{array}$$

then 2^*n*^ < *D* ^50/13^.

**Lemma 8 (see [10, 11])**

The equation

(12) $$\begin{array}{c} X^{2}+2^{m}=Y^{n},\;X,Y,m,n\in N,\;\;\text{ gcd }\left(X,Y\right)=1,\;\;\;n\geq 3 \end{array}$$

has only the solutions (*X*, *Y*, *m*, *n*) = (5,3, 1,3), (7,3, 5,4), and (11,5, 2,3).

**Lemma 9 (see [12])**

The equation

(13) X2−2m=Yn, X,Y,m,n∈N,  
gcd
(X,Y)=1, Y>1, m>1, n≥3

has only the solution (*X*, *Y*, *m*, *n*) = (71,17,7, 3).

**Lemma 10 (see [13])**

The equation

(14) $$\begin{array}{c} X^{m}-Y^{n}=1,\;X,Y,m,n\in N,\;\;\min\left\{X,Y,m,n\right\}>1 \end{array}$$

has only the solution (*X*, *Y*, *m*, *n*) = (3,2, 2,3).

## 3. Proof of Theorem

Let (*x*, *y*, *z*) be a solution of (2). If 2 | *y* and 2 | *z*, then we have *x* ≥ 3, *c* ^*z*/2^ + *b* ^*y*/2^ = 2^*x*−1^, and *c* ^*z*/2^ − *b* ^*y*/2^ = 2. It follows that

(15) $$\begin{array}{c} c^{z/2}=2^{x-2}+1,\;\;b^{y/2}=2^{x-2}-1. \end{array}$$

Applying Lemma 10 to (15), we can only obtain the solutions of types (i) and (ii).

If 2 | *y* and 2∤*z*, then we have

(16) $$\begin{array}{c} {\left(b^{y/2}\right)}^{2}+2^{x}=c^{z},\;2∤z. \end{array}$$

Applying Lemma 8 to (16), we can only get the solutions of types (iii), (iv), and (v).

Similarly, if 2∤*y* and 2 | *z*, using Lemmas 7 and 9, then we can only obtain the solutions of types (vi), (vii), and (viii). Finally, if 2∤*yz*, then the solutions are of type (ix). Thus, the theorem is proved.

## 4. Proof of Corollary

Since *c* = *b* + 2, (2) can be rewritten as

(17) $$\begin{array}{c} 2^{x}+b^{y}={\left(b+2\right)}^{z},\;x,y,z\in N. \end{array}$$

Let (*x*, *y*, *z*) be a solution of (17). By the theorem, (17) has only the solutions

(18) $$\begin{array}{c} \left(b,x,y,z\right)=\left(2^{r}-1,r+2,2,2\right),\;r\in N,\;\;r\geq 2 \end{array}$$

satisfying 2 | *y* and 2 | *z*.

If *x* = 1, 2∤*y* and 2 | *z*, then from (17) we get

(19) 2+by=2+((b+1)−1)y =1+(b+1)∑i=1y(−1)i−1(yi)(b+1)i−1=1+(b+1)∑j=1z(zj)(b+1)j−1=((b+1)+1)z=(b+2)z,

whence we obtain

(20) $$\begin{array}{c} y\equiv z\left(\mathrm{mod}\left(b+1\right)\right). \end{array}$$

But, since 2 | *b* + 1 and 2∤*y* − *z*, congruence (20) is impossible.

If *x* > 1, 2∤*y* and 2 | *z*, by the theorem, then we have *y* = 1 and 2^*x*^ < *b* ^50/13^. Hence, by (17), we get

(21) $$\begin{array}{c} b^{2}<{\left(b+2\right)}^{2}\leq {\left(b+2\right)}^{z}=2^{x}+b<b^{50/13}+b. \end{array}$$

Since *b* ≥ 3 and 2 | *z*, we see from (21) that *z* = 2. Substituting it into (17), we have *b* ^2^ + 3*b* − 4(2^*x*−2^ − 1) = 0 and

(22) $$\begin{array}{c} b=\frac{1}{2}\left(-3+\sqrt{2^{x+2}-7}\right). \end{array}$$

By (22), we get

(23) $$\begin{array}{c} b=\frac{1}{2}\left(X-3\right),\;\;x=n, \end{array}$$

where (*X*, *n*) is a solution of (10). Since 2∤*b* and *b* > 1, by Lemma 6, we can only have (*X*, *n*) = (181,13) and

(24) $$\begin{array}{c} \left(b,x,y,z\right)=\left(89,13,1,2\right), \end{array}$$

by (23).

If 2∤*yz*, then *b* ^*y*−1^ ≡ (*b* + 2)^*z*−1^ ≡ 1(mod⁡  8). Hence, by (17), we get 2^*x*^ ≡ (*b* + 2)^*z*^ − *b* ^*y*^ ≡ (*b* + 2) − *b* ≡ 2(mod⁡  8) and *x* = 1. It implies that the equation

(25) $$\begin{array}{c} \left(b+2\right)u^{2}-bv^{2}=2,\;u,v\in N \end{array}$$

has the solution

(26) $$\begin{array}{c} \left(u,v\right)=\left({\left(b+2\right)}^{\left(z-1\right)/2},b^{(y-1)/2}\right). \end{array}$$

Notice that the least solution of (25) is (*u*_1_, *v*_1_) = (1,1); *y* and *z* satisfy either *y* = *z* = 1 or min⁡{*y*, *z*} > 1. Applying Lemma 5 to (26), we only obtain that (*u*, *v*) = (1,1) and

(27) $$\begin{array}{c} \left(x,y,z\right)=\left(1,1,1\right). \end{array}$$

Thus, (17) has only the solutions (18), (24), and (27). The corollary is proved.
